# Strategies for Achieving Precision in Surgical Nursing Care—A Discursive Paper of a Project to Strengthen Person‐Centred Care, Teamwork and Professional Development

**DOI:** 10.1111/jan.70524

**Published:** 2026-02-02

**Authors:** Jenny Drott, Anna Forsberg, Karolina Härle

**Affiliations:** ^1^ Division of Nursing Sciences and Reproductive Health, Department of Health, Medicine and Caring Sciences Linköping University Linköping Sweden; ^2^ Clinical Department of Surgery in Linköping, Region Östergötland Linköping Sweden; ^3^ Institute of Health Sciences Lund University Lund Sweden; ^4^ Department of Cardiothoracic Surgery Skåne University Hospital, Lund University Lund Sweden

**Keywords:** interprofessional relations, nursing, patient care team, person‐centred care, programme implementation, staff development, surgical care

## Abstract

**Aim:**

To provide an overview of a project aimed at enhancing person‐centred surgical care through teamwork and professional development.

**Design:**

A discursive paper with the purpose of describing and discussing the project activities to strengthen person‐centred care, teamwork and professional development.

**Methods:**

This project aimed to strengthen evidence‐based surgical nursing care within a Surgery Department. The overarching goal was to foster an attractive and sustainable work environment for nursing professionals while simultaneously enhancing the quality of person‐centred care, thereby improving patient outcomes and safety. Ultimately, the project aimed to position the department as a leading example of excellence in surgical nursing care, where evidence‐based practice and person‐centred values form the foundation of everyday work.

**Results:**

The activities were guided by previous nursing evidence and aligned with Magnet Hospital standards. Continuous quality improvement efforts and team dialogue were central to achieving the goals. Leadership was provided by experienced nurses and researchers. The structured activities improved patient safety and care quality.

**Conclusions:**

The project was successful as it enhanced precision in surgical nursing care by implementing structured activities focused on person‐centred care, patient safety and professional development. These efforts led to improved quality of care and patient outcomes, demonstrating the effectiveness of evidence‐based practices.

**Impact and Implications for the Profession:**

A project with similar activities to those described in this paper can ensure that surgical patients receive high precision nursing care and serve as an example of promoting person‐centred surgical nursing.

**Reporting Method:**

None.

**Patient or Public Contribution:**

None.

## What Does This Paper Contribute to the Wider Global Clinical Community?

- The paper demonstrates how structured activities can significantly improve precision in surgical nursing care, providing a model for other healthcare institutions.
- By aligning with previous nursing evidence, the project showcases the importance of evidence‐based practices in achieving high‐quality and safe patient care.
- The paper highlights the critical role of continuous professional development and interdisciplinary teamwork for optimising clinical competencies and improving patient outcomes.

## 1 Introduction

A surgical department within a university hospital represents a complex, multiprofessional care environment that demands collaboration between healthcare professionals (HCPs) and patients to achieve high‐quality and safe care. There is a significant risk that prioritising efficiency may adversely affect patient safety. Research and professional nursing codes underscore the importance of adopting a more person‐centred approach (American Nurses Credentialing Center 2020; McCormack et al. 2002; McCance and McCormack 2025). The rapid pace of work and the high volume of surgical patients in inpatient care can result in unlicensed professional groups assuming tasks typically performed by licensed nursing professionals, potentially exposing the organisation to unnecessary challenges and leading to low precision in surgical nursing care. Pre‐ and post‐surgical care is predominantly focused on organ surveillance from a strictly medical perspective. The patient is therefore reduced to representing only objective disease data (disease‐oriented care), rather than an open system that enables them to be a person with an illness, able to tell their story and collaborate with professionals on care, treatment and rehabilitation plans, that is, person‐centred care (Ekman 2022). Surgical care is grounded in physiology and personalised medicine of the diseased organ or body. The therapies employed are based on genetic aspects or each patient's molecular, cellular or anatomical mechanisms. This objectified and biological view of surgical patients dominates the care system. However, it is a problematic view because it trivialises the necessary understanding of vulnerable patients (Kenward et al. 2017; Hynnekleiv et al. 2024). When each person is understood as unique, care actions can never be identical for each patient, despite the fact that the surgical procedure and associated treatment are the determining factors. Instead, daily clinical practice that includes person‐centred care must see the patient as both an object and a lived body. Surgical care practice can then be guided by person‐centred ethics and protection of the vulnerable person (Byrne et al. 2020; Moore et al. 2017). To ensure that surgical patients receive high precision nursing care, a project was initiated comprising various activities aimed at promoting person‐centred surgical nursing. This project was developed in accordance with current Swedish legislation, which emphasises person‐centred care and patient involvement (Sveriges Riksdag 2014).

## 2 Background

The project was initiated in response to a significant exodus of nurses in recent years, which resulted in the closure of care beds and an increased reliance on temporary nursing staff. In addition, the competence of assistant nurses has deteriorated, as many experienced individuals have retired or left the profession for other reasons. This reduction in skilled personnel among both nurses and assistant nurses has compromised the ability to maintain ‘world‐class care’, strong patient safety and high precision in surgical nursing, which are the goals of a university hospital. The project's objective was to enhance precision nursing and evidence‐based surgical nursing care within this challenging context. The implementation of evidence‐based surgical nursing interventions presents significant challenges, particularly when altering routine clinical practices to achieve excellence in surgical nursing in accordance with nursing core competencies. According to implementation science, the context in which these interventions are performed is crucial (Rogers et al. 2020). While nursing researchers are highly proficient at generating new evidence to inform healthcare, they are sometimes less effective at translating this new knowledge into clinical practice. Successful implementation of evidence‐based activities into practice is described as a function of the interplay of the following core elements: the nature of the evidence, the environment and the way in which the process is facilitated (Kitson et al. 1998).

A previous study provides evidence of evaluating knowledge translation, highlighting the challenges of translating research‐based knowledge into clinical practice (May et al. 2016). Hospital settings are often complex in the context of operationalising care processes. Complexity remains a significant barrier, despite the professional and ethical responsibility of organisations to contribute to person‐centred care, as well as the organisation and the profession, by means of new knowledge and improvements (American Nurses Credentialing Center 2020). Context plays a crucial role in the success of implementation efforts. It encompasses the unique circumstances and factors within which implementation takes place, such as the organisational culture and broader systemic influences. Strong leadership, a supportive environment and adequate resources enhance the success of an implementation. Innovation complexity occurs when the desired change in practice involves multiple steps, stakeholders and requires adequate actions across groups within the specific organisation (Dellenborg et al. 2019; Petticrew et al. 2013).

Existing practice norms, multidimensional professional relationships and diverse settings within an organisation increase this complexity (McCormack et al. 2002). Organisations are known to be complex environments for knowledge translation due to cultures and disciplinary norms (Novotná et al. 2012), and these factors may lead to an incomplete implementation process and a return to pre‐implementation behaviours (Nilsen and Bernhardsson 2019).

Different responses to organisational and workplace changes have been described in the literature, such as aggressive resistance, active resistance, passive resistance, indifference, support, involvement and commitment. Response to change is conceptualised as three components: cognitive, referring to opinions about changes; affective, referring to feelings about changes; and behavioural, referring to the actions taken (Coetsee 1999). Changes initiated by HCPs themselves are more likely to be accepted (Nilsen and Bernhardsson 2019).

A project to improve precision in evidence‐based surgical nursing through different strategies and activities to strengthen person‐centred care, teamwork and professional development was performed in a surgical department of a university hospital. These activities were guided by the European standard for patient involvement in healthcare (EN 17398:2020), which promotes respect for individual values and personalised care planning. By embedding these principles into practice, the project achieved higher quality of care and better patient outcomes, demonstrating both the impact of evidence‐based approaches and adherence to internationally recognised standards (Stjernswärd and Glasdam 2022). Results have shown that specialist surgical care nurses have gained increased nursing power/autonomy in the department, where greater responsibilities and the implementation of activities in small steps have been shown to be successful (Härle et al. 2025).

## 3 Data Sources

A detailed project description of activities to strengthen person‐centred surgical care, teamwork and professional development in order to provide an overview of the project.

## 4 Overview of the Issues

### 4.1 Theoretical Framework

Person‐centred care is an established concept in nursing and healthcare, with extensive literature detailing its key components (Byrne et al. 2020; McCance and McCormack 2025). The framework emphasises the importance of seeing each patient as a unique individual with their own needs and preferences. By means of shared decision‐making, a more engaging and respectful care environment is created within a partnership. Communication and collaboration between HCPs and patients are crucial for achieving the best possible care outcomes. Person‐centred care implies that HCPs consider the patient's life situation and social context, which can lead to higher precision in nursing. A core concept in person‐centred care is being taken seriously (Rantala et al. 2016). This concept can be measured (Forsberg and Rantala 2020) and includes eight different aspects: (1) The staff listened to me, (2) I received help to understand what has happened, (3) I received help to understand what is about to happen, (4) My concerns have been taken seriously, (5) My symptoms have been taken seriously, (6) I have been taken seriously as a person, (7) The staff made me feel good in the present moment and (8) The staff made me feel safe. Person‐centred care improves the patient's experience and satisfaction with care (Dalvindt et al. 2025). In addition, it can contribute to better health outcomes and reduced healthcare costs (Britten et al. 2020). Implementing person‐centred care requires training and support for HCPs. By fostering a culture of respect and empathy, healthcare can become more humane and effective.

### 4.2 Context

The initiative to strengthen evidence‐based surgical nursing care originated from the management of the clinic. The project was subsequently designed and led by two nurses from the clinic who had obtained a PhD, in collaboration with specialist nurses who acted as facilitators and were responsible for implementing various activities. The project received support from the management team and the head of the department.

This project aimed to strengthen evidence‐based surgical nursing care in a Surgery Department of a University hospital, with the goal of creating an attractive work environment for nursing professionals and strengthening person‐centred care.

The clinic's vision and goals are in line with the overall guidelines to be adhered to in person‐centred care and the intention to make the clinic/department an attractive workplace providing high quality and safe care. The project was influenced by previous nursing evidence in line with Magnet Hospital standards (Wilson et al. 2015; American Nurses Credentialing Center 2020). At the start of the project, a review of the various nursing roles in the clinic and their respective patient care responsibilities was conducted. Subsequently, a clear structure for how nursing care should be managed in the clinic was established. Several activities and strategies (e.g., prerequisites, education and organisational support) are required to translate evidence‐based surgical nursing into clinical practice.

## 5 Findings

### 5.1 Structure of Increased Precision in Evidence‐Based Surgical Nursing: How the Project Was Organised and Operationalised

The activities to strengthen person‐centred care, patient safety and development in the surgical nursing project were influenced by previous nursing evidence, expertise and excellent nursing knowledge from experienced nurses, as well as Magnet Hospital standards (Forsberg 2022, 2024; Wilson et al. 2015; American Nurses Credentialing Center 2020). The activities included continuous work to achieve quality goals, nursing quality and team dialogue. The project was led by two experienced nurses and researchers with great support from an excellent senior clinical Professor in nursing. Various activities related to person‐centred care, professional safety/development and teamwork were performed. Below, the activities are summarised in Table 1.

**TABLE 1:** Evidence‐based surgical nursing; activities in the project related to person‐centred care, professional safety/development and teamwork.

Evidence‐based surgical nursing			
	Person‐centred care	Teamwork	Professional safety/development
Activities	Head‐to‐toe assessment (HTTA) to foster precision	Clarify responsibilities between nurses and assistant nurses	Introduction‐programme for new nurses in the clinic based on the core competencies
	Nursing round (focus on person‐centred care and the patients' preferences and specific supportive care needs)	Create meeting places for collaboration in teams led by an Advanced Nurse Practitioner or Clinical Nurse Specialist	Clinical coach/mentor programme
	Nursing/individual care plans for targeted and goal‐oriented care	Collaboration between different groups in the clinic. HCPs in both inpatient and outpatient departments as well as in different disciplines	Supervision for newly graduated nurses, 2 h per month during the first 18 months
	Bedside‐rounds with focus on the specific patient situation, with the entire care team in dialogue with the surgical patient (all professionals in the care team)	Specific/One/A week with focus on nursing and the core competencies for the entire clinic and team‐members twice per year	Nursing Academy for the Clinic's Nurses to enhance evidence‐based nursing and professional discussion about research (e.g., journal clubs, presentations of important surgical nursing topics)
	Bedside reporting between shifts to achieve a safe and person‐centred handover with precision between shifts	Time allocated and scheduled for ethical discussions among the whole team	Professional development and strategic planning days for specialist nurses on 2 days per year
	Educational conversations, what is important for the patient, increased awareness of the patient's preferences and wishes for their care	Raises the team's awareness about the patient's needs and preferences	Understanding the patient's meaning‐ and sense making contributes to professional development. Gaining access to the patient's inside perspective fosters an understanding of the meaning of being a person in need of care
	A nurse with responsibility for patients in the form of a specialist nurse in the clinical ward from Monday–Friday	Invites the patient to become a valid partner in the health care team	Fosters the nurse–patient partnership

### 5.2 Person‐Centred Care

By following person‐centred care principles (please see the theoretical framework), HCPs can deliver more personalised and effective care, ultimately benefiting both individual patients and the healthcare system.

As a healthcare provider in this nursing project, embracing person‐centred care involves several key actions. We strive to:

- Recognise the person behind the medical diagnosis and surgical treatment/intervention. Treat each patient as a unique person with their own needs and preferences.
- Engage patients in their care decisions to create a more respectful and engaging environment.
- Maintain open communication and collaborate closely with patients to achieve the best supportive care outcomes.
- Consider the patient's life situation and social context to develop tailored nursing care plans.
- Strive for better health outcomes and reduced healthcare costs through person‐centred surgical nursing practices.
- Ensure HCPs receive the necessary training and support to implement person‐centred care in daily clinical practice.
- Foster a culture of respect and empathy to make surgical care more humane and effective.

#### 5.2.1 Head‐to‐Toe Assessment

We strive to achieve regular daily head‐to‐toe assessment (HTTA) in the wards. This comprehensive physical examination evaluates the status of the patient's body. This assessment is crucial for gathering detailed information about a patient's daily overall condition, which supports HCPs in the surgical department to formulate accurate nursing diagnoses and effective nursing care plans.

The first standard of practice that was implemented is that all patients should be examined by a nurse with warm hands on every shift. To promote trust and comfort, the careful and systematic HTTA is a cornerstone in precision surgical nursing (Fennessey and Wittmann‐Price 2011; Haugh 2019). In our context there has been a strong nursing tradition of interviewing the patient as a part of the nursing assessment, but this is not sufficient when the physical examination is missing. The nurse's gentle touch while performing the HTTA shows interest, thoroughness and the wish to take the patient seriously, constituting a person‐centred act. The HTTA enables identification of deviations from the norm or expected care pathway, thus risks or problems are detected at an early stage. The systematic approach is a part of the scientific method of collection and validation of patient data. Thus, evidence‐based nursing practice starts every time the nurse performs the HTTA, which provides an immediate overview of the patient's health status and wellbeing, replacing a time‐consuming reading of the patient's medical records. The nurse's analysis starts with the present, followed by advance planning based on the necessary prioritisation. In addition, the nurse supervises the team of assistant nurses to achieve precision nursing care.

#### 5.2.2 Nursing Round

The specific nursing round is a multiprofessional team meeting led by an Advanced Nurse Practitioner (ANP) or Clinical Nurse Specialist (CNS). The focus is nursing, that is, health promotion, relief of suffering or if relevant ensuring a dignified death. The nursing round is scheduled at the same time once a week and besides nurses, allied health professionals, for example, physiotherapists, occupational therapists, dieticians and social medical workers, participate. The surgeon is also invited. The ANP, or clinical nurse specialist, identifies patients in need of this specific nursing round, before which an educational conversation takes place with the patients. The aim of this conversation is to access the patient's sense‐ and meaning making (Nolvi et al. 2022) about their health situation and the perceived impact in everyday life, which enables the team to take the person seriously. The patients eligible for this round have had a complicated course of events or deviate from expected outcomes. Based on extended care needs the round focuses on the patient's risks, problems, resources, coping strategies and how the care team can address these challenges and strengthen the partnership and collaboration with the patient and relatives. A specific concern is whether there are challenges in the caring relationship. Following the nursing round, a nursing care plan for the patient is formulated or revised, detailing health problems, goals and interventions to achieve the goals. Nursing rounds were an important activity in the project, essential for ensuring high‐quality care and improving patient outcomes. To summarise, the key aspects of nursing rounds are:

- Regularly evaluating patients' physical, emotional, spiritual and social needs.
- Fostering open communication among the healthcare team to ensure coordinated care with the patient in focus.
- Involving patients in discussions about their care to ensure that their preferences and concerns are addressed.
- Working collaboratively with all members of the healthcare team to provide comprehensive care and using feedback from rounds to improve care practices and patient satisfaction.

By following these principles, nursing rounds support and create a more organised and responsive care environment that focuses on the patient, ultimately enhancing nursing precision and the quality of care.

#### 5.2.3 Nursing/Individual Care Plans

According to the Swedish Patient Act (Sveriges Riksdag 2014), as far as possible healthcare should be designed and implemented in consultation with the patient (Sveriges Riksdag 2014). An individualised nursing care plan is formulated by a CNS for all patients hospitalised for more than 5 days, who have complex care trajectories that do not follow an expected postoperative course or have been treated in an intensive care unit. The care plan is based on the patient's condition prior to the current illness and what the patient identifies as important in their life. The care plan is then updated daily by the responsible nurse.

#### 5.2.4 Bedside‐Rounds and Reporting Between Shifts

Focus is on the patient‐specific situation, with the entire care team in dialogue with the surgical patient. In the context of bedside rounds and shift reporting, it is imperative to focus on the patient‐specific situation. This approach ensures that the entire care team engages in a comprehensive dialogue with the surgical patient. Bedside rounds serve as a critical platform for multidisciplinary communication, where HCPs, including surgeons, nurses and other disciplines, collaborate to discuss the patient's current status, treatment plan and any emerging concerns. This method fosters a holistic understanding of the patient's condition, enabling the team to make informed decisions and provide tailored care. During shift reporting, the seamless transfer of information between outgoing and incoming staff is crucial. Detailed and accurate reporting ensures continuity of care, minimises the risk of errors and maintains the quality of patient care. By focusing on the patient's specific needs and circumstances, the care team can address any immediate issues and anticipate potential challenges, thereby enhancing patient outcomes. An important aspect of the handover between shifts is that the incoming and outgoing nurse and nurse assistant teams visit the patient together in order to review their goals of the day and assess intravenous and arterial lines, drainage and wounds. This is a particularly significant learning opportunity for new nurses and can reinforce their sense of control over the situation. In summary, emphasising patient‐specific situations during bedside rounds and shift reporting not only improves team communication and collaboration but also ensures that patient involvement is promoted and that the surgical patient receives the highest standard of care. Relatives have been central and important to involve and primarily involved based on the patient's wishes and preferences. In line with this, the attitudes of the staff have been investigated to strengthen the family perspective (Trulsson et al. 2023).

#### 5.2.5 Educational Conversations

All humans strive to know and understand to make sense of what is happening in their lives. When stricken by disease or illness, this meaning‐making process becomes more important (Forsberg 2024). Uncertainty is caused by unfamiliar symptoms, setbacks and complications, possibly leading to low self‐efficacy and poor self‐management (Almgren et al. 2017; Lindberg et al. 2020). To enable efficient and precise care planning, HCPs need access to the patient's meaning‐making. Also, as HCPs we should promote learning about the new health situation, for example, new medication, different treatments, necessary lifestyle or behavioural changes. The educational conversation is a tangible and constructive method for promoting mutual understanding and learning in the caring relationship (Öhlén and Friberg 2019). As the narrative is the core of person‐centred care, the first step in the nurse–patient encounter is to listen to the patient's narrative. This takes place when posing the question: ‘Please tell me how you understand and make sense of your current situation’. Possible follow‐up questions are: ‘*What do you prefer*? *What is important to you?*’

When the nurse has listened to the narrative the next question is: ‘*May I tell you a little about my professional understanding*?’ The goal of the educational conversation is not to deliver a standard set of information that might be overwhelming to the patient, but to establish a platform for mutual understanding. The first simple question stimulates reflection and thus experience‐based knowledge can be developed and exchanged. The HCPs learn about the meaning of being a care recipient and a suffering human being, while the patient increases their self‐awareness but also gains an understanding of the professionals' evidence‐based perspective. The educational conversation constitutes a solid base for person‐centeredness as it fosters mutual understanding. By creating space for the patient's personal models of understanding illness, it enables the nurse to promote a sense of coherence (Nolvi et al. 2022). When implementing this method, we focus on/listen to the patient's narrative and continuously stimulate the patient's learning process.

### 5.3 Teamwork

#### 5.3.1 Clarifying the Responsibilities of the Registered Nurse and the Assistant Nurse

Two groups of HCPs provide nursing to surgical patients. The assistant nurse graduated from high school, while the registered nurse (RN) graduated from university with a bachelor's degree in nursing science. The latter is an academic profession based on nursing theory, a code of ethics and current research for the provision of evidence‐based nursing care. The assistant nurse lacks scientific knowledge and is not trained in systematic patient assessments in accordance with the nursing process. Conflicts occurred in everyday clinical practice due to a perceived unclear mandate and invisible borders between these two groups. Thus, one important step in this project was to clarify the borders between the assistant nurse and the RN as well as establishing a clear framework for different nursing assignments. In the clinical setting, the roles and responsibilities of nurses and assistant nurses are now distinct yet complementary, ensuring efficient and effective patient care. Roles and responsibilities have been clarified, with laminated cards used to delineate roles in clinical practice. By clearly outlining these responsibilities, the healthcare team can function more effectively, ensuring that each member's skills are utilised to their fullest potential, ultimately enhancing the quality of patient care. Table 2 provides an overview of the roles and responsibilities.

**TABLE 2:** Overview of the responsibilities of registered nurses and assistant nurses.

Registered nurse	Assistant nurse
*Clinical assessment and diagnosis*:	*Basic patient care*:
Nurses are responsible for conducting comprehensive patient assessments, HTTA, vital signs, diagnosing health conditions, risks and developing individualised care plans	Assistant nurses provide basic patient care, including hygiene, dressing, feeding and assisting with mobility
*Medication administration*:	*Vital signs*:
Administers medications, monitors patient responses and manages any adverse effects	Records vital signs such as temperature, pulse, respiration and blood pressure
*Patient education*:	*Supportive tasks*:
Provides education to patients and their families regarding health conditions, treatments and prevention	Routine tasks, such as changing bed linen, cleaning patient areas and ensuring a safe patient environment
*Coordination of care*:	*Patient comfort*:
Coordinates care among various healthcare providers, ensuring seamless communication and continuity of care	Ensuring patient comfort, addressing immediate needs and providing emotional support
*Advanced procedures*:	*Communication*:
Performs advanced clinical procedures	Communicates patient needs and observations to the RN, contributing to the overall care plan
*Documentation*:	*Documentation*:
Maintains detailed and accurate patient records, documenting all aspects of care and treatment	Documenting parameters and aspects of care within their area of competence

#### 5.3.2 Team Collaboration

A goal in the project was effective collaboration, as the team is essential for delivering high‐quality patient care. Interdisciplinary teamwork fosters a holistic approach to patients, combining the expertise of various HCPs. This collaborative environment not only enhances patient outcomes, but also promotes professional growth and job satisfaction. Person‐centred care emphasises the importance of involving patients in their own treatment decisions. By establishing a partnership, it is possible to tailor care plans to better meet individual needs in accordance with our theoretical framework. This approach fosters a sense of empowerment, leading to improved patient satisfaction. Encouraging patients to express their concerns and participate in their care promotes a collaborative atmosphere where the patient's perspective is valued and integrated into the overall care strategy.

#### 5.3.3 ‘Nursing Weeks’

Our ‘Nursing Weeks’ were a learning support initiative to highlight nursing as a topic, necessary for achieving ‘world‐class care’. The nursing weeks were subsequently initiated and designed by two nurses (PhD) from the clinic, in collaboration with CNSs who acted as facilitators and were responsible for various activities. The choice of topics for the weeks has been made in collaboration to cover important topics in line with the clinic's nursing strategic areas. They took the form of a ‘conference in everyday clinical life’, where everyone was welcome to participate, even the patient in some parts, with everything in the immediate clinical environment. The weeks are dedicated periods aimed at nursing and typically feature a variety of activities, including lectures, workshops and seminars of topics of interest or priority. During ‘Nursing Weeks’, nurses can engage in continuing education, share best practices, evidence‐based nursing and receive acknowledgment for their contributions to patient care. These events not only boost morale but also reinforce the importance of nursing in achieving optimal health outcomes.

### 5.4 Professional Safety and Development

Professional security during the first year of nursing was identified as a crucial focus area due to the unit's difficulties in recruiting and retaining new nurses. Being a newly graduated nurse can involve both mental and physical exhaustion as well as ethical stress (See et al. 2023; Odland et al. 2014). The newly graduated nurse needs support, and one way is a transition programme led by experienced nurses (CNS) (See et al. 2023).

Within this focus area, activities were introduced, including: (1) an introductory programme for new nurses during their first year featuring regular professional meetings led by a CNS, including one administrative day per month, (2) mentor programme administrative days, and (3) regular reflection meetings with managers focusing on the work environment.

#### 5.4.1 Introductory Programme for Newly Graduated Nurses

The introductory programme based on the nursing core competencies for newly recruited nurses encompasses several key components designed to support their transition and professional development. Clinical orientation involves hands‐on training under the supervision of a mentor and theoretical education with days consisting of lectures and practical exercises to build foundational knowledge. Each new nurse is supported by an experienced clinical coach who provides personalised support and guidance. The coach, who has at least 5 years of experience at the clinic, meets with the new nurse regularly during the first year. Time is also allocated for individualised in‐depth learning of departmental routines, shadowing other professionals and visiting the operating room. The professional introductory programme is led by experienced specialist nurses and comprises several meetings, which serve as platforms for reflection and discussion on daily work and nursing core competencies, aimed at enhancing professional confidence.

#### 5.4.2 Specialist Nurse Days

Given the vital role of experienced nurses in ensuring robust operations and the importance of encouraging them to remain in the workplace, it was deemed essential to introduce activities specifically targeting these nurses (Jakobsson et al. 2023). The project implemented professional meetings, consisting of full‐day sessions held twice a year for the clinic's specialist nurses. These meetings focused on career development, nursing leadership, implementation and other topics aligned with the specialist nurses' areas of expertise. Additionally, the clinic already had a nursing council and a nursing academy, which held approximately five meetings per year. All nurses at the clinic were invited to participate in these academically focused activities, such as journal clubs.

## 6 Discussion

This project aimed to enhance the quality of healthcare delivery by focusing on three key areas: person‐centred care, teamwork and professional development. The key areas intended to enhance strategies to achieve precision in surgical nursing care led to many positive outcomes, particularly in terms of clarifying evidence‐based surgical nursing. The overarching goal was to foster an attractive and sustainable work environment for nursing professionals. Successful implementation of nursing rounds and the introduction programmes for new nurses were notable achievements, although some activities require further refinement. We observed several positive effects during our project, where all activities encompassed both development and professional pride in conducting evidence‐based nursing with focus on the best interests of our patients. Meaningful work is an important driving force that motivates specialist nurses to remain within the surgical department (Drott et al. 2023; Jakobsson et al. 2023). Positive outcomes in the current project increased the feeling of meaningful work in the team with focus on the patient needs. Missed Nursing Care (MNC) is a global phenomenon, defined as any aspect of required patient care that is either omitted or delayed. Ball et al. (2018) emphasise that adequate nurse staffing is crucial for reducing MNC and improving the survival of surgical patients. The study provides strong evidence that MNC is not merely a workforce issue but is directly linked to patient outcomes (Ball et al. 2018). Evidence also suggests that the main reason for MNC is inadequate number of staff and a significant association between MNC and nurses' intention to leave their workplace. To achieve safe person‐centred fundamental care a warranted investment in nursing within the organisation is necessary (Edfeldt et al. 2024). The current nursing project implemented strategies and interventions aimed at reducing nurse attrition, optimising competence utilisation and strengthening the delivery of fundamental care. The activities were designed to enhance meaningfulness in nursing practice and are therefore expected to reduce the risk of MNC. The HTTA, nursing rounds, individual care plans and educational conversations constitute important tools for identifying nursing needs and for developing structured plans to implement nursing interventions that might otherwise be deprioritized or overlooked, thereby minimising the occurrence of MNC.

Educational conversations with patients play a crucial role in healthcare by fostering mutual understanding and learning between professionals and patients. This process is particularly important for patients facing a severe disease or illness, as it helps them make sense of their situation and manage the uncertainty caused by unfamiliar symptoms and complications (Forsberg 2024; Almgren et al. 2017; Lindberg et al. 2020). By engaging in person‐centred educational conversations, professionals can access the patient's thoughts and meaning‐making process, which is essential for precise care planning and promoting patient self‐efficacy (McCance and McCormack 2025). This aspect was perceived as very challenging by some professionals during the project, often due to a lack of time. Lack of time increases the risk of MNC, which necessitates that organisational leadership establish strategies to ensure the provision of safe and high‐quality care (Edfeldt et al. 2024). There may also be barriers to asking such open questions and listening to the patient's narrative, which require education and training. The narrative approach is central to person‐centred care, where the initial step involves listening to the patient's story. This method allows professionals to understand the patient's perspective and preferences, facilitating the formulation of a tailored care plan.

This part of the project probably requires more work in the future because it not only support patients' development of experience‐based knowledge, but also enables professionals to learn about the patient's experience of illness and suffering. It promotes a sense of coherence and mutual understanding, both of which are fundamental to person‐centred care (Öhlén and Friberg 2019; Nolvi et al. 2022). Implementing educational conversations in the surgical context ensures that the patient's narrative remains central, continuously stimulating their learning process and enhancing the overall quality of care.

Professional security during the first year of practice is a critical area, particularly due to the challenges in recruiting and retaining new nurses. Newly graduated nurses often face significant mental and physical exhaustion, as well as ethical stress (See et al. 2023; Odland et al. 2014; Demir et al. 2024). To address these challenges, support mechanisms such as transition programmes led by experienced nurses are essential (See et al. 2023).

In the project several activities were introduced to support new nurses. These included an introductory programme during their first year, featuring regular professional meetings led by a CNS, one administrative day per month, mentor programme administrative days and regular reflection meetings with managers focusing on the work environment. These initiatives aimed to provide structured support and enhance the professional security of newly graduated nurses. We have noted in evaluations (presented elsewhere) that the transition from nursing education to clinical work has been strengthened in several ways. The introduction programme aimed to strengthening the nurse's professional role and may serve as a strategy to reduce the risk of MNC. This can be achieved by highlighting and reinforcing the areas where MNC is most common, such as oral care, mobilisation and repositioning (Edfeldt et al. 2024). These professional meetings also enhance the nurse's leadership in collaboration with assistant nurses, which is particularly important since it is often the assistant nurse, or the nurse together with the assistant nurse, who performs the fundamental nursing interventions.

Additionally, we acknowledge the importance of retaining experienced nurses, as some of the activities implemented in the project focused on their professional development. The specific initiatives and sessions were designed to align with the CNSs areas of expertise and encourage their continued engagement in the workplace (Jakobsson et al. 2023; Härle et al. 2025). Overall, these structured activities aimed to enhance both the professional security of newly graduated nurses and the professional development of experienced nurses, contributing to a more robust and supportive nursing environment. To enhance nursing practice, it can be essential to establish clearly defined clinical roles for RN/PhDs as highlighted by previous evidence. Despite the positive impact of active leadership, ongoing and robust efforts are necessary to achieve further advances (Orton et al. 2022). It may be valuable for the overall organisation to invest in academic positions for nurses to establish robust structures that can contribute to evidence‐based nursing in a long‐term perspective.

## 7 Conclusions

The project successfully enhanced precision in surgical nursing care by implementing structured activities focused on person‐centred care, patient safety and professional development. These efforts were aligned with the European standard for patient involvement in healthcare (EN 17398:2020), which emphasises respect for patient values and individualised care planning. By integrating these principles, the project achieved improved quality of care and patient outcomes, demonstrating the effectiveness of evidence‐based practices and compliance with internationally recognised guidelines.

## Author Contributions

**Jenny Drott:** conceptualization, methodology, visualisation, writing original draft – review and editing. **Anna Forsberg:** conceptualization, methodology, visualisation, writing – review and editing: **Karolina Härle:** conceptualization, methodology, visualisation, writing – review and editing.

## Funding

The authors have nothing to report.

## Conflicts of Interest

The authors declare no conflicts of interest.

## Data Availability

The data that support the findings of this study are available from the corresponding author upon reasonable request.
